# *Bessera elegans* (Asparagaceae): Botany, Phytochemistry, and Cytotoxic and Insecticidal Activities of an Underexplored Mexican Species

**DOI:** 10.3390/molecules31061030

**Published:** 2026-03-19

**Authors:** Luz Janet Tagle-Emigdio, David Osvaldo Salinas-Sánchez, Erubiel Toledo-Hernández, Miguel Angel Mendoza-Catalán, Ana Elvira Zacapala-Gómez, Daniel Hernández-Sotelo, Anette Guadalupe Leyva-Bello, Edgar Jesús Delgado-Nuñez, Rodolfo Figueroa-Brito, César Sotelo-Leyva

**Affiliations:** 1 Facultad de Ciencias Químico-Biológicas (FCQB), Universidad Autónoma de Guerrero (UAGro), Av. Lázaro Cárdenas S/N, Col. La Haciendita, Chilpancingo 39087, Guerrero, Mexico; 15170@uagro.mx (L.J.T.-E.); erubieltoledo@gmail.com (E.T.-H.); mamendoza@uagro.mx (M.A.M.-C.); 17757@uagro.mx (A.E.Z.-G.); dhernandez@uagro.mx (D.H.-S.); 18295622@uagro.mx (A.G.L.-B.); 2 Centro de Investigación en Biodiversidad y Conservación (CIByC), Universidad Autónoma del Estado de Morelos (UAEM), Avenida Universidad 1001, Chamilpa, Cuernavaca 62210, Morelos, Mexico; 3 Escuela de Estudios Superiores del Jicarero, Universidad Autónoma del Estado de Morelos (UAEM), Carretera Galeana-Tequesquitengo s/n, Colonia El Jicarero, Jojutla 62909, Morelos, Mexico; 4 Facultad de Ciencias Agropecuarias, Pecuarias y Ambientales, Universidad Autónoma de Guerrero (UAGro), Iguala 40040, Guerrero, Mexico; edgarjezus@gmail.com; 5 Centro de Desarrollo de Productos Bióticos (CEPROBI-IPN), Carr Yautepec—Jojutla s/n-km. 6.5, San Isidro 62739, Morelos, Mexico; rfigueroa@ipn.mx

**Keywords:** cancer, medicinal plants, phytochemistry, extracts

## Abstract

*Bessera elegans* (Asparagaceae) is an endemic Mexican species that is traditionally valued for ornamental purposes and locally reported medicinal uses, yet it remains largely underexplored from phytochemical and biological perspectives. The identification of bioactive secondary metabolites from under-investigated plant species is a key step toward developing plant-derived compounds with potential biotechnological applications. Therefore, in this context, we compile and critically analyze the available information on the botany, phytochemistry, and reported cytotoxic and insecticidal activities of *B. elegans*. Phytochemical studies, mainly focused on the bulbs, have led to the isolation of steroidal glycosides, homoisoflavonoids, flavonoids, and norlignans. Several of these compounds exhibit cytotoxicity against human cancer cell lines, including leukemia and lung adenocarcinoma models. More recent investigations of flower extracts have revealed additional classes of secondary metabolites and preliminary insecticidal activity, highlighting the species’ chemical diversity. Although the current biological evidence remains limited, the reported cytotoxic and insecticidal effects provide a biochemical basis supporting the relevance of *B. elegans* as a potential source of plant-derived bioactive compounds. This review highlights existing knowledge gaps and emphasizes the need for further phytochemical and biological studies to support future biotechnological applications of metabolites from underexplored endemic plant species.

## 1. Introduction

Mexico is recognized as one of the world’s most megadiverse countries due to its extraordinary biological richness, which arises from the complex interactions among its varied topography, wide range of climates, and diverse ecosystems [1]. It is estimated that Mexico harbors between 10 and 12% of global biodiversity and is one of the nations with the highest diversity of vascular plants [2]. Floristic studies indicate that the country’s vascular flora comprises approximately 23,000 to 26,000 species, a substantial proportion of which are endemic [3].

Within this botanical context, the family Asparagaceae (order Asparagales) comprises a diverse group of monocotyledonous plants with a wide geographical distribution. Its members are mainly perennial herbs, many of which are geophytes bearing rhizomes, bulbs, or tubers as storage organs. Morphologically, they are characterized by simple leaves with parallel venation, generally actinomorphic flowers with six tepals and six stamens, and a superior trilocular ovary. The fruits are berries or capsules, and the seeds are often coated with phytomelanin [4,5]. In addition to their morphological diversity, several Asparagaceae species are known for their rich secondary metabolite profiles, particularly steroidal saponins. These are considered characteristic constituents of the family and have been associated with a wide range of biological activities, including cytotoxic, anti-inflammatory, antimicrobial, and insecticidal effects [6,7]. The family includes numerous taxa of ornamental, nutritional, and phytochemical relevance, highlighting its biological and biotechnological importance. Within this remarkable plant diversity, the genus Bessera is a distinctive biological component of the family Asparagaceae, representing a valuable endemic element of the Mexican flora [8].

*Bessera elegans* Schult. f. (Asparagaceae) is commonly known as “coral drops” or “earrings,” due to the distinctive morphology of its inflorescences, which are characterized by pendulous, tubular flowers reminiscent of small lanterns or delicate ornamental pendants [9]. This species is endemic to Mexico, with a distribution restricted to the western and central regions of the country, where it occurs mainly in open habitats associated with pine–oak forests, grasslands, and ecotonal zones [10]. *Bessera elegans* is frequently found in disturbed environments, particularly along the margins of agricultural fields and in areas of secondary vegetation, suggesting a certain degree of ecological adaptability to anthropogenic landscapes. Such habitats expose the species to diverse biotic pressures such as herbivory and insect predation. These pressures could contribute to the biosynthesis of defensive secondary metabolites, such as steroidal saponins, which are widely reported in monocotyledonous plants and members of the family Asparagaceae [11].

Although *B. elegans* is primarily valued for ornamental purposes due to the striking morphology and intense coloration of its pendulous inflorescences, traditional knowledge from various regions of Mexico attributes additional functional uses to this species. In particular, *B. elegans* has been reported to possess insecticidal properties and is locally employed in domestic pest control practices, especially in rural contexts, as documented in ethnobotanical studies [12,13].

Despite its ornamental value and traditional use in some regions of Mexico, *Bessera elegans* remains a poorly studied species from chemical and biological perspectives. The available literature indicates that studies on its secondary metabolites and bioactive properties are still scarce. Research conducted mainly on the plant bulbs has demonstrated cytotoxic and pro-apoptotic activity against various human cancer cell lines, with these effects primarily attributed to steroidal glycosides isolated from the extracts [14]. In addition, the flavonoids present in the species exhibit pronounced cytotoxicity, particularly against leukemia cancer cells, suggesting a relevant pharmacological potential [15]. Nevertheless, the lack of comprehensive studies that jointly address the botany, phytochemistry, and cytotoxic and insecticidal activities of *B. elegans* highlights the need for updated reviews that consolidate the existing knowledge. In this context, the present review aims to synthesize, contextualize, and critically analyze the available information, identify knowledge gaps, and highlight priority research directions for future studies.

## 2. Botany

### 2.1. Taxonomical Classification

The genus *Bessera* was named in honor of the botanist Dr. Wilibald Swibert Joseph Gottlieb von Besser, a prominent professor of botany during the nineteenth century. *Bessera elegans* Schult. f. belongs to the monocotyledonous lineage, and its current taxonomic placement is supported by recent morphological and phylogenetic evidence [9]. The taxonomic classification scheme of the species is summarized in Table 1.

In addition, *B. elegans* exhibits a geophytic growth habit, a characteristic trait of several members of Brodiaeoideae that confers adaptive advantages in temperate and seasonally variable environments. Geophytes are a relevant component of the Mexican flora, particularly in mountainous regions of central Mexico, where climatic seasonality and topographic complexity favor their diversification and persistence through underground storage organs [16].

### 2.2. Morphological Characteristics and Distribution

**Figure 1:**
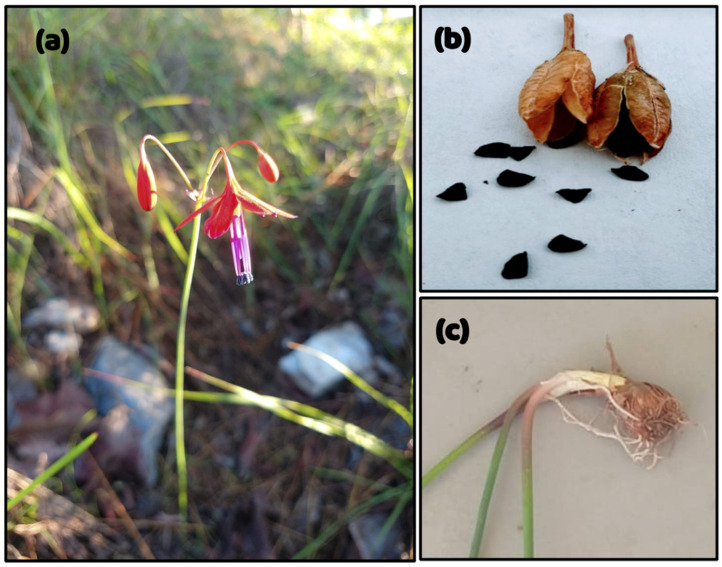
Morphology of *Bessera elegans*. (**a**) *Bessera elegans* flower in situ. (**b**) Dry capsules and mature seeds of the species, and (**c**) bulbs and roots.

*Bessera elegans* (Figure 1) is a perennial geophytic plant belonging to the subfamily Brodiaeoideae (Asparagaceae) and is endemic to Mexico. It produces erect flowering stems that can reach approximately 60 cm in height, with basal herbaceous leaves. The flowers are arranged in umbelliform inflorescences, generally composed of three to nine flowers, which display predominantly scarlet coloration, with tonal variation ranging from deep red to purplish hues. The species is broadly distributed in western and central Mexico, extending from the Sierra Madre Occidental (Durango and Sinaloa), across the Trans-Mexican Volcanic Belt, to the Sierra Madre del Sur (Oaxaca). These morphological traits and distribution patterns are consistent with the taxonomic circumscription and morphological variation described for the genus Bessera, as supported by recent morphological and phylogenetic evidence [9,17].

Despite the available taxonomic and morphological descriptions, ecological and population-level studies on *Bessera elegans* remain scarce. Future research addressing its ecological interactions, population dynamics, and adaptive traits could provide valuable insights into the evolutionary and ecological significance of this endemic species.

## 3. Phytochemistry

### 3.1. Phytochemical Overview of the Asparagaceae Family

The Asparagaceae family is recognized as a rich source of structurally diverse secondary metabolites, particularly steroidal saponins, which are considered chemotaxonomic markers of the family [18]. These compounds, predominantly spirostanols and furostanols, have been isolated from various genera, including Asparagus, Ophiopogon, Polygonatum, and Dracaena, and exhibit a wide array of biological activities, including cytotoxic, anti-inflammatory, antimicrobial, and immunomodulatory effects [19].

Beyond steroidal saponins, Asparagaceae species produce other significant classes of secondary metabolites. Homoisoflavonoids, for instance, are characteristic of the subfamily Hyacinthoideae (e.g., Scilla, Eucomis) and have demonstrated notable cytotoxic and anti-angiogenic properties [20]. Flavonoids (e.g., myricetin and quercetin derivatives) and norlignans have also been reported, contributing to the family’s chemical diversity [21]. The biological significance of Asparagaceae metabolites is well-documented. For example, steroidal glycosides from *Asparagus cochinchinensis* have shown potent cytotoxic activity against human cancer cell lines through apoptosis induction and cell cycle arrest [20]. Similarly, compounds such as proscillaridin A, isolated from plants of the genus Scilla (Asparagaceae), have been reported to inhibit topoisomerase I and II activity at nanomolar concentrations, a key mechanism in cancer chemotherapy [22]. This rich phytochemical and pharmacological background provides a compelling rationale for investigating underexplored genera within the family, such as Bessera.

### 3.2. Compounds Identified in the Bulbs of *Bessera elegans*

**Figure 2:**
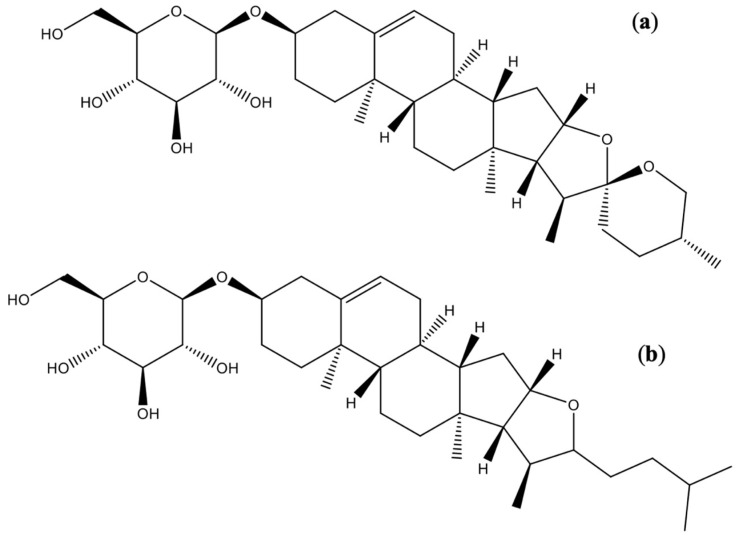
Structures of steroidal glycosides isolated from the bulbs of *Bessera elegans*. (**a**) Spirostanol-type glycoside; (**b**) furostanol-type glycoside.

Within the chemical and pharmacological diversity of the Asparagaceae family, the genus Bessera represents a particularly interesting, yet scarcely studied, case. Despite limited exploration of *B. elegans* phytochemistry to date, available studies have already detected secondary metabolites of considerable biological interest that align with the chemotaxonomic profile of the family while also displaying unique structural features. To date, research has focused mainly on the bulbs (modified stems that grow underground and function as storage organs), from which several bioactive compounds have been isolated. In particular, the phytochemical study of *B. elegans* bulbs conducted by Matsuo et al. [14] led to the isolation and identification of 14 steroidal glycosides. Of these compounds, nine (1–9) corresponded to novel structures and five (10–14) had been previously reported (Table 2). Collectively, these metabolites are mainly classified as spirostanol and furostanol glycosides (Figure 2).

In subsequent studies, Matsuo et al. [15] investigated the phytochemistry of *B. elegans* bulbs and isolated and identified seven phenolic compounds, including both novel structures and previously known metabolites. Among the most structurally relevant compounds, two new homoisoflavonoids were identified: (3R)-5,7-dihydroxy-6-methyl-3-(3′-hydroxy-4′-methoxybenzyl) chroman-4-one, and (3R)-5,7,3′-trihydroxy-4′-methoxy-6-methylspiro [2H-1-benzopyran-7′-bicyclo [4.2.0] octa [1,3,5] trien]-4-one. The latter was characterized by the presence of the uncommon scillascillin-type skeleton. The presence of this rare skeleton in *B. elegans* is particularly noteworthy from a chemotaxonomic perspective, as scillascillin-type homoisoflavonoids are typically considered characteristic markers of the subfamily Hyacinthoideae within Asparagaceae [21], suggesting a closer phytochemical relationship than previously recognized. In addition, three known flavonoids (myricetin, annulatin, and cannabiscitrin) and two known norlignans (nyasol and 3′-methoxynyasol) were reported. Table 3 summarizes the phenolic compounds identified in the bulbs of *B. elegans.*

### 3.3. Phytochemical Composition of Flower Extracts of Bessera elegans

The available phytochemical evidence on flower extracts of *B. elegans* obtained by sequential maceration reveals strong effects of solvent polarity and analytical technique on the composition of secondary metabolites isolated. Non-polar fractions are dominated by saturated hydrocarbons, long-chain aliphatic alcohols, and steroids. In contrast, the acetone fraction concentrates structurally more complex metabolites, including polyphenolic compounds, aglycones, and terpenoids, which are detected as well-defined chromatographic peaks in HPLC analyses. These results, reported by Sotelo-Leyva et al. [23], are comparatively summarized in Table 4. Although several classes of secondary metabolites have been identified in *B. elegans*, phytochemical investigations remain limited to a small number of plant organs and studies. Further research integrating metabolomic approaches and bioactivity-guided fractionation is required to better characterize the chemical diversity of this species and its potential biological functions.

## 4. Biological Activity of *Bessera elegans*

Phytochemical and biological studies within the genus Bessera remain extremely limited. Most of the available research has focused on *B. elegans*. In contrast, other species of the genus, such as *Bessera tuitensis*, *Bessera elegantissima*, and *Bessera ramirezii*, remain largely unexplored from chemical and pharmacological perspectives. Consequently, comparative data on secondary metabolites and biological activities within the genus are currently scarce. In this context, although information on the biological activity of *B. elegans* remains limited, available studies indicate that this species exhibits significant bioactivity, particularly in cytotoxic and insecticidal activities.

These activities have been mainly evaluated using extracts and isolated compounds from different plant organs and are closely related to the diversity of secondary metabolites identified. Therefore, this section summarizes the main findings from the literature on the cytotoxic and insecticidal activities of *B. elegans.*

### 4.1. Cytotoxic Activity

The cytotoxic activity of *B. elegans* has been investigated primarily through studies focused on compounds isolated from its bulbs, with particular emphasis on steroidal glycosides and phenolic metabolites. From an ecological perspective, the accumulation of these bioactive steroidal glycosides in underground storage organs such as bulbs is consistent with a chemical defense strategy against belowground herbivores (e.g., nematodes, insect larvae) and soil-borne pathogens, a functional role widely documented for saponins in geophytic plants [7,11,24]. Therefore, the potent cytotoxic effects observed against human cancer cell lines may reflect the inherent biological reactivity of metabolites evolved to interact with cellular targets in competing organisms.

To date, the most comprehensive and systematic evaluations have been conducted by Matsuo et al. [14], who isolated nine new and five previously known steroidal glycosides and assessed their cytotoxic effects against human tumor cell lines, including HL-60 (promyelocytic leukemia) and A549 (lung adenocarcinoma), using normal human fibroblasts (TIG-3) as a reference for non-selective toxicity. Among the compounds evaluated, a pseudo-furostanol glycoside exhibited pronounced cytotoxic activity, inducing time-dependent apoptosis in both HL-60 and A549 cells and causing cell cycle arrest at the G0/G1 phase in A549 cells. These findings suggest that steroidal glycosides from *B. elegans* may exert cytotoxic effects by modulating apoptosis-related pathways and regulating the cell cycle. A comparative summary of the cytotoxic activities reported for these compounds is presented in Table 5.

Subsequent work by Matsuo et al. [15] expanded the phytochemical and biological characterization of *B. elegans* through the isolation of seven phenolic compounds, including homoisoflavonoids, flavonoids, and norlignans. These metabolites were evaluated mainly against the HL-60 cell line and, in general, displayed lower cytotoxic potency than the steroidal glycosides. Within this group, homoisoflavonoids exhibited the most notable activity, whereas flavonoids and norlignans, such as nyasol and its methoxylated derivative, showed weak or marginal inhibition of cell viability (Table 6). These results are consistent with reports from other members of Asparagaceae and related monocotyledonous taxa, in which steroidal glycosides are frequently identified as the primary contributors to cytotoxic and antiproliferative effects. At the same time, phenolic compounds often play a secondary or modulatory role [7,11,24].

From a broader perspective, steroidal saponins and glycosides have been reported to exhibit cytotoxic and pro-apoptotic activities across a wide range of cancer cell lines, via mechanisms including membrane permeabilization, mitochondrial dysfunction, and activation of caspase-dependent pathways [25,26]. The bioactivity profiles observed for *B. elegans* thus align well with the established pharmacological behavior of steroidal metabolites from monocotyledonous plants. However, despite these promising results, cytotoxicity studies on *B. elegans* remain limited in scope, focusing on a small number of cell lines and isolated compounds. Further investigations integrating additional cancer models, mechanistic assays, and comparative analyses of different plant organs are required to fully elucidate the cytotoxic potential and therapeutic relevance of this species.

### 4.2. Insecticidal Activity

The available information on the insecticidal activity of *B. elegans* is limited and is based on only two reports. The first account is by Cruz-Duque et al. [10], who report the empirical use of *B. elegans* for insecticidal purposes in some regions of Mexico; however, that study does not include experimental evaluations, controlled bioassays, or quantitative data to assess the species’ insecticidal efficacy. The presence of insecticidal compounds in the flowers of *B. elegans* seems ecologically relevant, given that the species inhabits disturbed ecosystems with high biotic pressure. In this context, the biosynthesis of defensive metabolites in reproductive structures could thus constitute an adaptive strategy to mitigate damage caused by specialized herbivores that prey on reproductive tissues, thereby increasing the biological fitness of the species in ecologically challenging environments [27,28].

The most relevant study to date, and currently the only one to systematically evaluate the insecticidal potential of *B. elegans*, was conducted by Sotelo-Leyva et al. [23]. Their study analyzed flower extracts obtained using solvents of different polarity (n-hexane and acetone). The study evaluated their insecticidal effects against the aphid *Melanaphis sacchari* Zehntner (Hemiptera: Aphididae), the main pest of sorghum (*Sorghum bicolor* L. Moench). The bioassays included contact toxicity and artificial feeding models and found dose- and time-dependent insecticidal activity that varied significantly depending on extract polarity and the route of exposure, as summarized in Table 7.

The predominance of long-chain hydrocarbons and aliphatic alcohols in the non-polar floral extracts of *B. elegans* is of particular biological interest, as similar lipid profiles have been described as key structural components of the cuticle in aphid species. Brey et al. [29] reported that the cuticular lipids of the aphid *Acyrthosiphon pisum* are mainly composed of long-chain hydrocarbons and *n*-alcohols, including nonacosane (C_29_), triacontane (C_30_), hentriacontane (C_31_), hexadecanol (C_16_), eicosanol (C_20_), and docosanol (C_22_). Using electron microscopy, these authors demonstrated that such lipids are integral components of the epicuticle and procuticle, and that their extraction with organic solvents compromises the integrity of the integument. The chemical similarity between the non-polar constituents of *B. elegans* floral extracts and aphid cuticular lipids provides an important biochemical basis for the contact toxicity observed in insecticidal bioassays.

Further support for this hypothesis comes from studies on other long-chain lipophilic compounds. In aphids exposed to long-chain fatty acids, symptoms such as abdominal contractions, dehydration, and tissue necrosis have been reported, which have been associated with alterations of the cuticle and cellular desiccation processes [30]. These observations suggest that lipophilic compounds of this type may affect the epicuticular wax layer and contribute to contact toxicity.

On the other hand, the polar extracts of *B. elegans*, particularly those obtained with acetone, contain flavonoids and other phenolic compounds that may contribute to insecticidal activity through distinct mechanisms. Flavonoids have been widely reported to exhibit insecticidal activity against several aphid species, including *Aphis gossypii*, *Acyrthosiphon pisum*, *Myzus persicae*, and *Rhopalosiphum padi*, by interfering with key physiological processes such as feeding, growth, development, and reproduction [31]. These metabolites have been shown to inhibit digestive enzymes, disrupt neuronal signaling, and impair detoxification mechanisms, ultimately leading to insect mortality [32]. Taken together, these findings suggest that the insecticidal activity of *B. elegans* may be associated with the complementary actions of non-polar and polar metabolites, each acting through distinct physiological mechanisms.

Overall, the limited number of biological studies highlights the need for further investigations integrating phytochemical characterization, toxicological evaluation, and mechanistic assays to clarify the biological potential of *Bessera elegans* metabolites and their possible applications in pharmacology and sustainable pest management.

## 5. Conclusions

This review synthesizes available information on *B. elegans*, an endemic Mexican species of the family Asparagaceae, primarily valued for its ornamental potential and limited ethnobotanical use. Despite its biological relevance, important gaps remain in the understanding of its phytochemical composition and associated bioactivities, indicating that *B. elegans* is still an underexplored species. Phytochemical studies have focused mainly on the bulbs, leading to the identification of flavonoids, homoisoflavonoids, and steroidal glycosides with demonstrated cytotoxic activity. More recent investigations on flower extracts have revealed additional metabolites and promising insecticidal effects.

Overall, *B. elegans* represents a dual source of bioactive compounds with biotechnological potential. Future studies should focus on isolating the phenolic compounds responsible for the insecticidal activity in flower extracts, evaluating the synergy between polar and non-polar extracts, and elucidating the cytotoxic molecular mechanisms of bulb glycosides.

## Figures and Tables

**Table 1:** Taxonomic classification of *Bessera elegans*.

Kingdom:	Plantae
Subkingdom:	Tracheobionta
Superdivision:	Spermatophyta
Division:	Magnoliophyta
Class:	Liliopsida
Subclass:	Liliidae
Order:	Asparagales
Family:	Asparagaceae
Subfamily:	Brodiaeoideae.
Genus:	Bessera
Species:	*Bessera elegans* Schult. f

Taxonomic classification based on Chase et al. [5].

**Table 2:** Steroidal glycosides in the bulbs of *Bessera elegans*.

ID	Type	Formula	Structure	Sugars
1	Spirostanol	C_56_H_92_O_28_	2α-hydroxylated spirostane tetraglycoside with a branched sugar chain	4
2	Spirostanol	C_50_H_82_O_22_	2α,3β,14α-trihydroxy spirostane tetraglycoside	4
3	Spirostanol	C_58_H_96_O_31_	Oxygenated spirostane tetraglycoside; extended sugar chain	4
4	Spirostanol	C_54_H_88_O_27_	2α,14α,27-trihydroxy spirostane glycoside with galactose, xylose, and glucose	4
5	Spirostanol	C_58_H_92_O_30_	12-oxo spirostane pentaglycoside containing arabinose	5
6	Furostanol	C_56_H_94_O_30_	26-O-β-D-glucosyl 2α,22α-dihydroxy furostane pentaglycoside	5
7	Furostanol	C_56_H_94_O_30_	Furostanol analogue with a similar oligosaccharide pattern	5
8	Furostanol	C_56_H_94_O_30_	Furostanol oligoglycoside structurally related to compounds 6–7	5
9	Furostanol	C_62_H_98_O_32_	Furost-20(22)-en pentaglycoside containing xylose, rhamnose, and arabinose	5
10	Spirostanol	n.d.	Spirostane triglycoside with arabinose, xylose, glucose, rhamnose, and galactose	5
11	Spirostanol	n.d.	2α-hydroxy spirostane tetraglycoside; galactose–xylose–glucose–galactose sequence	4
12	Furostanol	n.d.	26-O-β-D-glucosyl 2α,3β,22α-trihydroxy furostane aglycone; monoglucoside	1
13	Furostanol	n.d.	26-O-β-D-glucosyl 22α-hydroxy furostane tetraglycoside containing galactose, xylose, glucose	4
14	Furostanol	n.d.	26-O-β-D-glucosyl 2α,22α-dihydroxy furostane tetraglycoside; galactose–xylose–glucose chain	4

n.d. = not reported.

**Table 3:** Phenolic compounds isolated from the bulbs of *Bessera elegans*.

Class	Name	Structural Type
Homoisoflavonoid	(3R)-5,7-dihydroxy-6-methyl-3-(3′-hydroxy-4′-methoxybenzyl) chroman-4-one	New homoisoflavonoid
Homoisoflavonoid	(3R)-5,7,3′-trihydroxy-4′-methoxy-6-methylspiro [2H-1-benzopyran-7′-bicyclo [4.2.0] octa [1,3,5] trien]-4-one	New scillascillin-type homoisoflavonoid
Flavonoid	Myricetin	Known flavonoid
Flavonoid	Annulatin	Known flavonoid
Flavonoid	Cannabiscitrin	Known flavonoid
Norlignan	Nyasol	Known norlignan
Norlignan	3′-Methoxynyasol	Known norlignan

**Table 4:** Phytochemical composition of *Bessera elegans* flower extracts according to extract type and analytical technique.

Extract	Analysis	Number of Compounds	Chemical Classes	Representative Compounds
*n*-Hexane (non-polar)	GC–MS	26 compounds (99.76% of the extract)	Saturated hydrocarbons, aliphatic alcohols, and steroids	Triacontane (21.01%), 1-heptacosanol (18.82%), nonacosane (12.41%)
Acetone (intermediate polarity)	HPLC	13 major peaks	Polyphenols, flavonoid aglycones, and terpenes	Polyphenols (peaks 1–4), aglycones (peaks 5–9), terpenes (peaks 10–13)

GC–MS: Gas chromatography–mass spectrometry; HPLC: High-performance liquid chromatography.

**Table 5:** Cytotoxic activity of steroidal glycosides isolated from the bulbs of *Bessera elegans*.

Compound/Group	Class	Cell Lines	Cytotoxic Effect
Pseudo-furostanol glycoside	Steroidal glycoside	HL-60, A549, TIG-3	Induces time-dependent apoptosis in HL-60 and A549 cells; causes cell cycle arrest at the G0/G1 phase in A549 cells
Other steroidal glycosides	Steroidal glycosides	HL-60, A549, TIG-3	Variable, structure-dependent cytotoxic activity; reduced effects in normal cells

**Table 6:** Cytotoxic activity of phenolic compounds isolated from the bulbs of *Bessera elegans*.

Compound	Class	Cell Lines	Cytotoxic Effect
(3R)-5,7-dihydroxy-6-methyl-3-(3′-hydroxy-4′-methoxybenzyl) chroman-4-one	Homoisoflavonoid	HL-60	Detectable cytotoxic activity
(3R)-5,7,3′-trihydroxy-4′-methoxy-6-methylspiro [2H-1-benzopyran-7′-bicyclo [4.2.0]octa [1,3,5]trien]-4-one	Homoisoflavonoid	HL-60	Moderate cytotoxic activity
Myricetin	Flavonoid	HL-60	Limited activity
Annulatin	Flavonoid	HL-60	Limited activity
Cannabiscitrin	Glycosylated flavonoid	HL-60	Weak activity
Nyasol	Norlignan	HL-60	Weak activity
3′-Methoxynyasol	Norlignan	HL-60	Weak activity

**Table 7:** Summary of the insecticidal activity of *Bessera elegans* extracts against *Melanaphis sacchari*.

Extract	Polarity	Major Component	Route of Exposure	Mortality (72 h, 10,000 ppm)	Suggested Mechanism
n-hexane	Low	Long-chain hydrocarbons	Contact	88%	Affinity for the aphid cuticle (external toxicity)
Acetone	Intermediate	Polyphenolic compounds	Artificial diet	69%	Cellular or digestive damage following ingestion (internal toxicity)

## Data Availability

No new data were created or analyzed in this study. Data sharing is not applicable to this article.

## Abbreviations

The following abbreviations are used in this manuscript:

CG-MS	Gas Chromatography–Mass Spectrometry
HPLC	High-Performance Liquid Chromatography
HL-60	Human promyelocytic leukemia cell line
A549	Human lung adenocarcinoma cell line
TIG-3	Normal human fibroblast cell line
ppm	Parts per million
